# The clinicopathological relevance of uniform CD56 expression in anaplastic large cell lymphoma: a retrospective analysis of 18 cases

**DOI:** 10.1186/s13000-020-01059-y

**Published:** 2021-01-05

**Authors:** Bao-Hua Yu, Yan Zhang, Tian Xue, Ruo-Hong Shui, Hong-Fen Lu, Xiao-Yan Zhou, Xiong-Zeng Zhu, Xiao-Qiu Li

**Affiliations:** 1 Department of Pathology, Fudan University Shanghai Cancer Center, 270 Dong-an Road, Xuhui District, Shanghai, 200032 China; 2 Department of Oncology, Shanghai Medical College, Fudan University, Shanghai, 200032 China

**Keywords:** Anaplastic large cell lymphoma, CD56 expression, ALK, Differential diagnosis, Overall survival

## Abstract

**Background:**

Anaplastic large cell lymphoma (ALCL) with uniform CD56 expression is a rare condition, that has been described in limited literature, and its clinicopathological features have not yet been well illustrated. The aim of our study was to fully investigate the clinical, histological, immunohistochemical and molecular features of CD56+ ALCL.

**Methods:**

The clinical and histological characteristics of CD56+ ALCL cases were retrospectively evaluated. The immunohistochemical phenotype, status of Epstein-Barr virus (EBV) and T-cell receptor (TCR) gene rearrangement were examined. Overall survival was also analyzed.

**Results:**

Eighteen (5.8%) cases with diffuse CD56 expression were identified out of 313 archived ALCL cases with CD56 test. CD56 expression was significantly higher in ALK+ systemic ALCLs (sALCLs) (13/64, 20.3%) than in ALK- sALCLs (3/101, 3.0%) (*p* < 0.001) as well as primary cutaneous ALCLs (2/148, 1.4%) (*p* < 0.001). Regarding the CD56+ ALK+ sALCLs, the median age was 20 years (range, 8–60 years) with a male-to-female ratio of 2.3:1, and these cases more frequently affected extranodal sites (11/38, 28.9%) rather than lymph nodes (2/26, 7.7%) (*p* = 0.038). Eleven (84.6%) cases presented with stage I-II diseases, which was significantly more than their CD56- ALK+ counterparts (45.5%) (*p* = 0.015). Histologically, 2 ALK+ cases were of small cell variant and all the others displayed characteristic morphology of classic ALCL. Regarding the immunophenotype, both CD30 and CD56 were diffusely positive in all cases. CD3, CD43, anaplastic lymphoma kinase-1 (ALK1), TIA-1, EMA expression was observed in 30.8% (4/13), 90.9% (10/11), 100% (13/13), 100% (9/9), and 80.0% (8/10) cases, respectively. EBV infection was consistently absent. Monoclonal TCR gene rearrangement was found in 100% (5/5) of investigated ALK+ cases. Chemotherapy with a CHOP regimen was most frequently employed. The 3-year overall survival (OS) rate for CD56+ ALK+ patients was 92.0%, compared with 73.0% for their CD56- counterparts, but there was no significant difference in OS between the two groups (*p* = 0.264).

**Conclusions:**

Uniform CD56 expression is an unexpected condition in ALCL. Of ALK+ ALCLs, CD56 expression correlated with a high frequency of early stage and an extranodal predominance. It is of great importance to raise awareness of this condition and familiarity with its characteristic features to avoid diagnostic and therapeutic pitfalls. Further investigations are warranted for a better understanding of this unusual phenotype and the significance of CD56 expression in ALCL.

## Background

Anaplastic large cell lymphoma (ALCL) is a rare distinct type of peripheral T-cell lymphoma. Typical ALCL is characterized by the proliferation of generally large atypical lymphocytes with pleomorphic, horseshoe−/kidney-shaped or doughnut-like nuclei and abundant cytoplasm, according to the World Health Organization (WHO) classification of tumors of hematopoietic and lymphoid neoplasms [1, 2]. Systemic ALCL (sALCL) is currently subdivided into anaplastic lymphoma kinase positive (ALK +) ALCL and ALK negative (ALK-) ALCL; in addition, primary cutaneous ALCL (pcALCL) and breast-implant associated ALCL are also recognized as separate subtypes of ALCL [1–3]. CD30 is consistently positive in each subtype of ALCL, as one of its diagnostic criteria.

CD56, a 140-kd protein derived from alternative splicing of the gene-encoding neural cell adhesion molecule, is frequently expressed by natural killer (NK) cells. CD56 appears to be a useful marker for extranodal NK/T-cell lymphoma, nasal type (ENKTL), as its expression is almost consistent [4]. CD56 has also been reported in other lymphoid neoplasms, including a subset of peripheral T-cell lymphomas, monomorphic epitheliotropic intestinal T-cell lymphomas, hepatosplenic T-cell lymphomas, some types of B-cell lymphoma and some cases of acute myeloid leukemia [5, 6]. Although CD56 expression was not thought to occur in ALCL initially, it has subsequently been documented in ALCL in both adults and children, in both nodal and extranodal sites [5, 7–10]. However, reports on CD56+ ALCL are extremely limited, and the clinicopathological relevance of CD56 expression in ALCL is poorly illustrated. Dunphy et al. reviewed nine cases with CD56+ ALCL confined to children, including both primary cutaneous cases and systemic cases, and ALK status was unclear in some cases [7]. Suzuki et al. highlighted the prognostic significance of CD56 expression in sALCLs, in which ALK+ and ALK- cases were analyzed as a whole [5]. Moreover, CD56 expression in different subtypes of ALCL has never been elucidated in detail. In addition, due to the unexpected occurrence and overlapping features, CD56+ ALCL is frequently misdiagnosed as other CD56+ lymphomas, resulting in inappropriate treatment. Therefore, increased awareness of its potential occurrence and familiarity with its characteristic features are important for avoiding both diagnostic and therapeutic pitfalls.

Herein, we described a series of ALCL cases with diffuse and strong expression of CD56 and fully analyzed their histopathologic characteristics, immunophenotype, and molecular features as well as their survival for the purpose of enhancing the recognition of this phenomenon, which might be imperative for not only an accurate diagnosis but also a subsequent optimal treatment.

## Methods

### Case selection

Eighteen patients with ALCL with uniform CD56 expression diagnosed between 2009 and 2019 were collected from the archival files of the Department of Pathology, Fudan University Shanghai Cancer Center (Shanghai, P.R. China), all of which were consultation cases. The study was approved by the Institutional Review Board of Fudan University Shanghai Cancer Center (Shanghai Cancer Center Ethical Committee). All tumors were histologically reviewed and the diagnosis was confirmed by two experienced hematopathologists (BHY and XQL) according to the WHO classification of tumors of the hematopoietic and lymphoid tissues [1, 2]. Cases showing CD56 positivity in only a subpopulation of tumor cells were excluded. The clinical information and follow-up data were also collected.

### Histology and immunohistochemistry

Formalin-fixed paraffin-embedded tissue blocks were available for all cases and recut for routine hematoxylin and eosin (H&E) staining and the immunohistochemical procedure. Histological characteristics were examined by two experienced hematopathologists (BHY and XQL). Immunohistochemical studies were performed on these tissue sections, using a Ventana BenchMark Ultra Autostainer (Ventana Medical System Inc., Roche Tucson, AZ, USA) and the Ventana ultraView Universal DAB Detection Kit. The primary antibodies included cytoplasmic CD3, CD4, CD8, CD20, CD30, CD43, CD56, T-cell-restricted intracellular antigen-1 (TIA-1), ALK1, EMA and Ki67. All of these antibodies were commercial products from Roche Ventana. All stainings were performed with appropriate positive and negative controls.

### In situ hybridization (ISH) study for EBV-encoded small RNA (EBER)

The status of EBV infection was assessed by ISH detection for EBER on paraffin-embedded tissue sections using fluorescein-labeled oligonucleotide probes (INFROM EBER Probe, Ventana), as previously described [11]. The visualization system used was the BenchMark XT with enzymatic digestion (ISH Protease 2, Ventana) and the iVIEW Blue v3 Detection Kit (Ventana). A known EBV-positive tumor was used as a positive control in each run. An appropriate negative control section was also included.

### T-cell receptor (TCR) gene rearrangement analysis

Genomic DNA was extracted from formalin-fixed paraffin-embedded tissue sections, and TCR-β, γ and δ gene rearrangements were detected by polymerase chain reaction (PCR) assays. Amplifiability of the DNA was confirmed by concurrent PCR amplification of the β-globin sequence. Each PCR study was carried out in duplicate and included positive, negative, and no-template controls. The PCR products were analyzed by capillary electrophoresis, using the ABI PRISM 310 Genetic Analyzer (Applied Biosystems, CA, USA).

### Statistical analysis

Overall survival (OS) time was defined as the interval from the initial diagnosis to the date of death from any cause or the last contact. OS was estimated using the Kaplan-Meier method. Categorical variables were compared by the χ2 test, and measurement data were analyzed using Pearson correlation analysis. All the statistical analyses were performed using the SPSS software package (SPSS version 25.0; Inc., Chicago, IL, USA). *P* values of less than 0.05 were considered statistically significant.

## Results

### Clinical features

We identified 18 cases with diffuse and uniform expression of CD56 out of 313 cases with CD56 investigation in our database, and thus, the frequency was approximately 5.8%. The clinical features of these 18 patients are summarized in Table 1. In detail, 13 cases (72.2%) were diagnosed as ALK+ sALCL, 3 (16.7%) were ALK- sALCL (case no. 4, 14, 18), and 2 (11.1%) were pcALCL (case no. 2 and 13). CD56 expression was more frequently observed in ALK+ sALCLs (13/64, 20.3%) than in ALK- sALCLs (3/101, 3.0%) or pcALCLs (2/148, 1.4%) (both *p* < 0.001). Given that the cases in both the ALK- sALCL and pcALCL groups were quite limited, we mainly focused on the ALK+ sALCL group when analyzing the clinicopathological features and survival.

**Table 1:** The clinical features of patients with CD56+ ALCL in the current study

Case No.	Age (y)/Sex	Sites of involvement	Stage	B symptoms	LDH level	Treatment	Status	Follow-up time (months)
1	19/M	Soft tissue of waist	IE	No	Normal	CT	Alive	49
2	63/M	Skin of right lower extremity; right inguinal lymph nodes	IIE	No	Normal	CT (CHOP)	Alive	51
3	60/M	Right anterior inferior gingiva	IE	No	Normal	CT (CHOP)	Alive ^a^	69
4	52/F	Left iliac bone	NA	NA	NA	NA	NA	NA
5	13/F	Left breast	IE	No	Normal	CT (CHOP)	Alive	77
6	20/M	Parathoracic soft tissue	IE	No	Normal	CT (CHOP)	Alive	79
7	25/F	Right breast	IE	No	Normal	CT + RT	Alive	86
8	26/M	Left axillary soft tissue	IE	Yes	NA	CT (CHOP) + RT	Alive	92
9	23/M	Right cervical lymph nodes	I	Yes	NA	CT (CHOP)	Alive	37
10	35/M	Cervical lymph node	I	Yes	Normal	CT (CHOP) + RT	Alive	104
11	15/M	Nasopharynx, posterior nares; masses on the back, left thigh and left axilla	IVE	Yes	Normal	CT (CHOP) + RT	Alive	114
12	17/M	Nasopharynx, oropharynx, posterior nares; cervical lymph nodes	IIE	Yes	Normal	L-Asparaginase +DICE	Dead	0.5
13	47/M	Skin lesions of left leg, left thigh and right leg	IVE	No	Normal	CT + RT+ ASCT	Alive	97
14	67/M	Multiple lymphadenopathy of neck, axilla, subclavian and retroperitoneal; tumorous pleural fluid and ascites	IV	No	high	refusal to treatment	Dead	22
15	27/M	Soft tissue of right thigh root	IE	No	NA	CT (CHOP)	Alive	40
16	8/F	Right nasopharynx, right upper neck, the anterior area of the right ear	IIE	NA	Normal	CT	Alive	95
17	19/F	Soft tissue of the right chest wall, abdominal wall and left groin	IVE	No	NA	NA	Alive	1
18	22/M	Left cervical lymph nodes	NA	Yes	NA	NA	Dead	1

^a^ Recurrence 24 months after original diagnosis

*CT* chemotherapy, but regimen unavailable, *CT (CHOP)* chemotherapy with cyclophosphamide, doxorubicin, vincristine and prednisone, *RT* radiation therapy, *DICE* chemotherapy with dexamethasone, ifosfamide, cisplatin and etoposide, *ASCT* autologous stem cell transplantation, *NA* not available

Among the 13 ALK+ patients, 9 were male and 4 were female, with a male-to-female ratio of 2.3:1, and there was no significant difference in sex distribution between CD56+ and CD56- ALK+ sALCLs (*p* = 0.706). The median age was 20 years (mean, 23.6 years; range, 8–60 years), which was similar to that of CD56- ALK+ patients (median, 23 years; mean, 29.4 years; range, 5–74 years) (*p* = 0.427) (Table 2).

**Table 2:** The clinicopathological relevance of CD56 expression in ALK+ sALCLs

	Total (%)	CD56+ group (%)	CD56- group (%)	***p*** value
**Sex**	64	13	51	0.706
Male	47 (73.4)	9 (69.2)	38 (74.5)	
Female	17 (26.6)	4 (30.8)	13 (25.5)	
**Age**	64	13	51	0.427
Median	23	20	23	
Range	5–74	8–60	5–74	
**Stage**	46	13	33	0.015^*^
I-II	26 (56.5)	11 (84.6)	15 (45.5)	
III-IV	20 (43.5)	2 (15.4)	18 (54.5)	
**Site**	64	13	51	0.038^*^
Nodal	26 (40.6)	2 (15.4)	24 (47.1)	
Extranodal	38 (59.4)	11 (84.6)	27 (52.9)	
**B symptoms**	37	12	25	0.308
With	20 (54.1)	5 (41.7)	15 (60.0)	
Without	17 (45.9)	7 (58.3)	10 (40.0)	

^*^ Statistically significant *p* values are in bold

Nine cases (69.2%) in the current CD56+ series presented with stage I disease, 2 (15.4%) with stage II disease and 2 (15.4%) with stage IV disease. The stage of CD56+ ALK+ sALCLs was significantly earlier than that of CD56- ALK+ sALCLs (*p* = 0.015). B symptoms were observed in 5 (41.7%) out of 12 patients. With regard to the anatomic sites of involvement, 2 cases occurred in lymph nodes and 11 originally presented in the extranodal sites, including the upper aerodigestive tract (*n* = 3), soft tissue (*n* = 5), breast (*n* = 2), and primary uncertainty (*n* = 1), with the latter case involving both the nasopharynx and distant soft tissue at diagnosis. CD56 positivity was much more frequently seen in extranodal cases (11/38, 28.9%) than in nodal cases (2/26, 7.7%) (*p* = 0.038) (Table 2).

### Histopathology

**Fig. 1:**
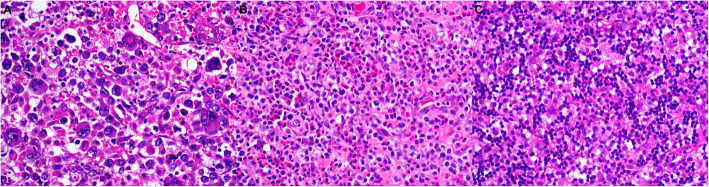
Representative morphology of CD56+ ALCLs in our series. **a** This tumor manifested obvious pleomorphism in morphology, and both typical hallmark cells and large cells with multiple nuclei in a wreath-like pattern were observed (H&E, × 400). **b** Atypical neoplastic cells admixed with a large number of eosinophils (H&E, × 400). **c** This small cell variant ALCL consisted predominantly of small- to medium-sized lymphoid cells with hyperchromatic nuclei, mimicking histiocytoid or plasmacytoid cytomorphology, and more irregular larger cells were scattered (H&E, × 400)

Microscopically, 16 cases exhibited typical morphology of ALCL, composed mainly of large atypical cells with a moderate to abundant amount of basophilic or acidophilic cytoplasm. Characteristic hallmark cells were frequently seen with eccentric, horseshoe- or kidney-shaped nuclei. Cells with multilobular nuclei or multinucleated cells, some in a wreath-like pattern, can be found in 2 of these cases (Fig. 1a). Brisk mitotic figures were observed in each case. Inflammatory infiltration consisting of small lymphocytes and plasma cells was present in variable proportions, and a large number of eosinophilic granulocytes were seen in one case (Fig. 1b).

The remaining two cases in ALK+ group were of the small-cell variant, which consisted predominantly of monomorphic small- to medium-sized lymphoid cells, with hyperchromatic and slightly irregular nuclei (Fig. 1c). Nucleoli were inconspicuous. Some neoplastic cells exhibited histiocytoid or plasmacytoid cytomorphology, mimicking reactive lymphoid cells or histiocytes. A few slightly larger and more irregular cells with an eccentrically located kidney-shaped nucleus resembling those of hallmark cells were scattered.

### Immunohistochemical findings

**Fig. 2:**
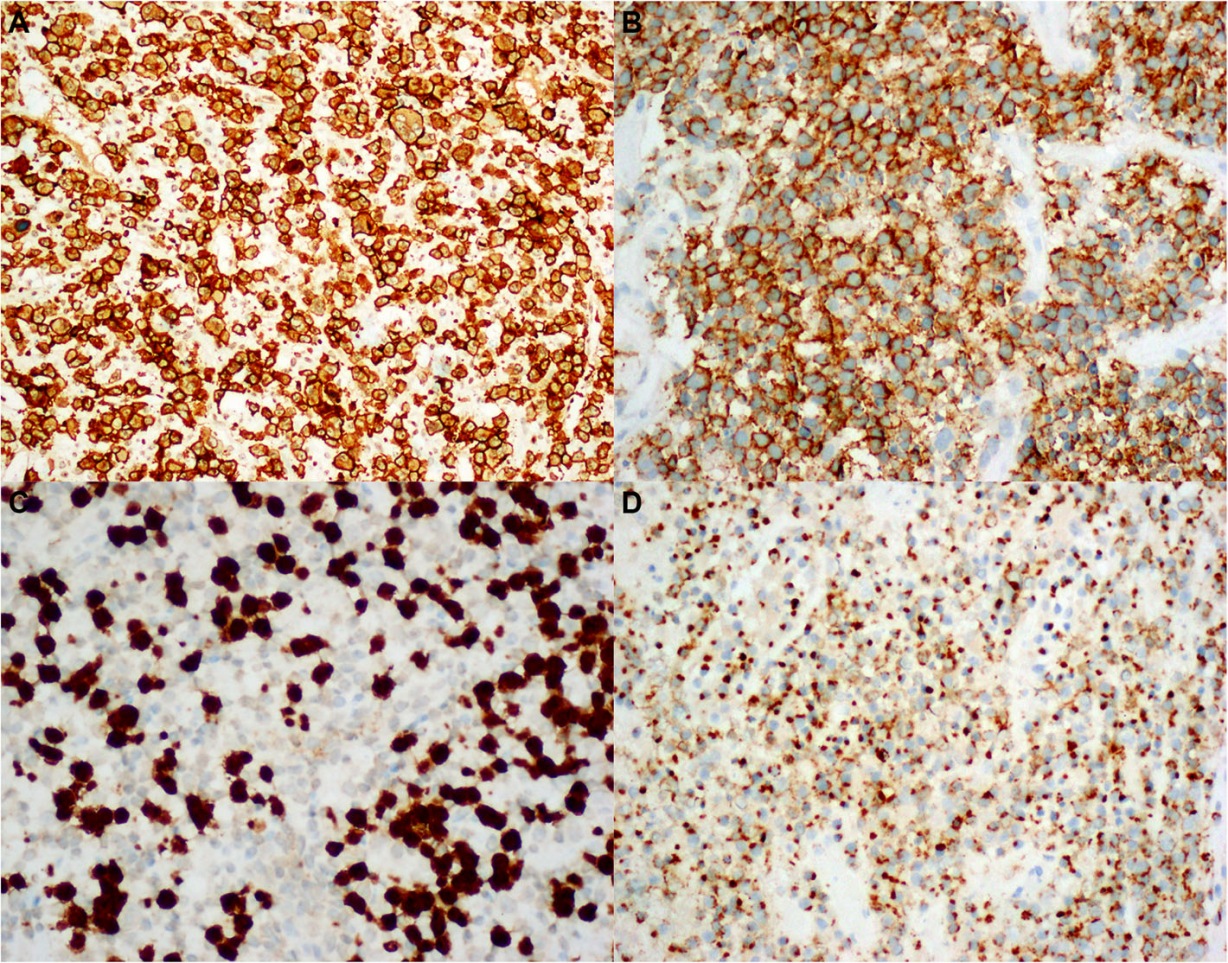
Representative images of immunohistologic staining of selected markers. Strong and uniform staining for CD30 (**a**) and CD56 (**b**) was observed in each case; and tumor cells were positive for ALK1 (**c**) and TIA-1 (**d**) in some cases (× 400)

The immunohistochemical findings are summarized in Table 3 for all 18 cases. Of the 13 CD56+ ALK+ sALCLs, CD3 was positive in 4 cases (30.8%), whereas CD43 expression was found in 90.9% cases (10/11). Both CD30 (13/13, 100%) and CD56 (13/13, 100%) were constantly positive in a diffuse and uniform pattern in each case. TIA-1 staining was observed in 100% (9/9), and EMA was observed in 80.0% (8/10) of the tested tumors, including 3 with focal immunoreactivity. CD4 and CD8 were positive in 60.0% (3/5) and 40.0% (2/5) of cases, respectively. CD20 expression was consistently absent (Fig. 2).

**Table 3:** Summary of the immunohistochemical and EBER-ISH findings of the CD56+ ALCLs in the current study

Case No.	Age (y)/Sex	CD20	CD3	CD4	CD8	CD30	CD43	CD56	ALK1	TIA-1	EMA	Ki67	EBER
1	19/M	–	–	ND	ND	+	+	+	+	ND	+	70%	–
2	63/M	–	+	–	–	+	ND	+	–	+	–	80%	–
3	60/M	–	–	ND	ND	+	ND	+	+	+	ND	70%	–
4	52/F	–	+	+	+	+	+	+	–	+	ND	90%	–
5	13/F	–	–	+	–	+	+	+	+	ND	Few+	90%	–
6	20/M	–	–	+	–	+	ND	+	+	+	+	80%	–
7	25/F	–	+	–	+	+	+	+	+	+	ND	70%	–
8	26/M	–	–	ND	ND	+	+	+	+	ND	+	70%	–
9	23/M	–	+	ND	ND	+	+	+	+	+	–	50%	–
10	35/M	–	–	ND	ND	+	+	+	+	+	+	80%	–
11	15/M	–	–	ND	ND	+	+	+	+	+	ND	70%	–
12	17/M	–	Few+	+	–	+	+	+	+	+	–	80%	–
13	47/M	–	–	+	–	+	ND	+	–	ND	ND	80%	–
14	67/M	–	+	+	ND	+	+	+	–	–	–	80%	–
15	27/M	–	Few+	–	+	+	+	+	+	+	Few+	80%	–
16	8/F	–	–	ND	ND	+	–	+	+	ND	+	50%	–
17	19/F	–	–	ND	ND	+	+	+	+	+	+	80%	–
18	22/M	–	Few+	ND	ND	+	+	+	–	+	–	80%	–

*M* male, *F* female, *ND* not done

### ISH for EBER, and TCR gene rearrangement

ISH detection for EBER revealed that no positive signals were found in tumor cells in all cases. Five CD56+ ALK+ cases underwent TCR gene rearrangement detection and they all exhibited monoclonal TCR gene rearrangement, with the same phenotype of TCR-γ+/β+/δ-.

### Treatment and outcome

**Fig. 3:**
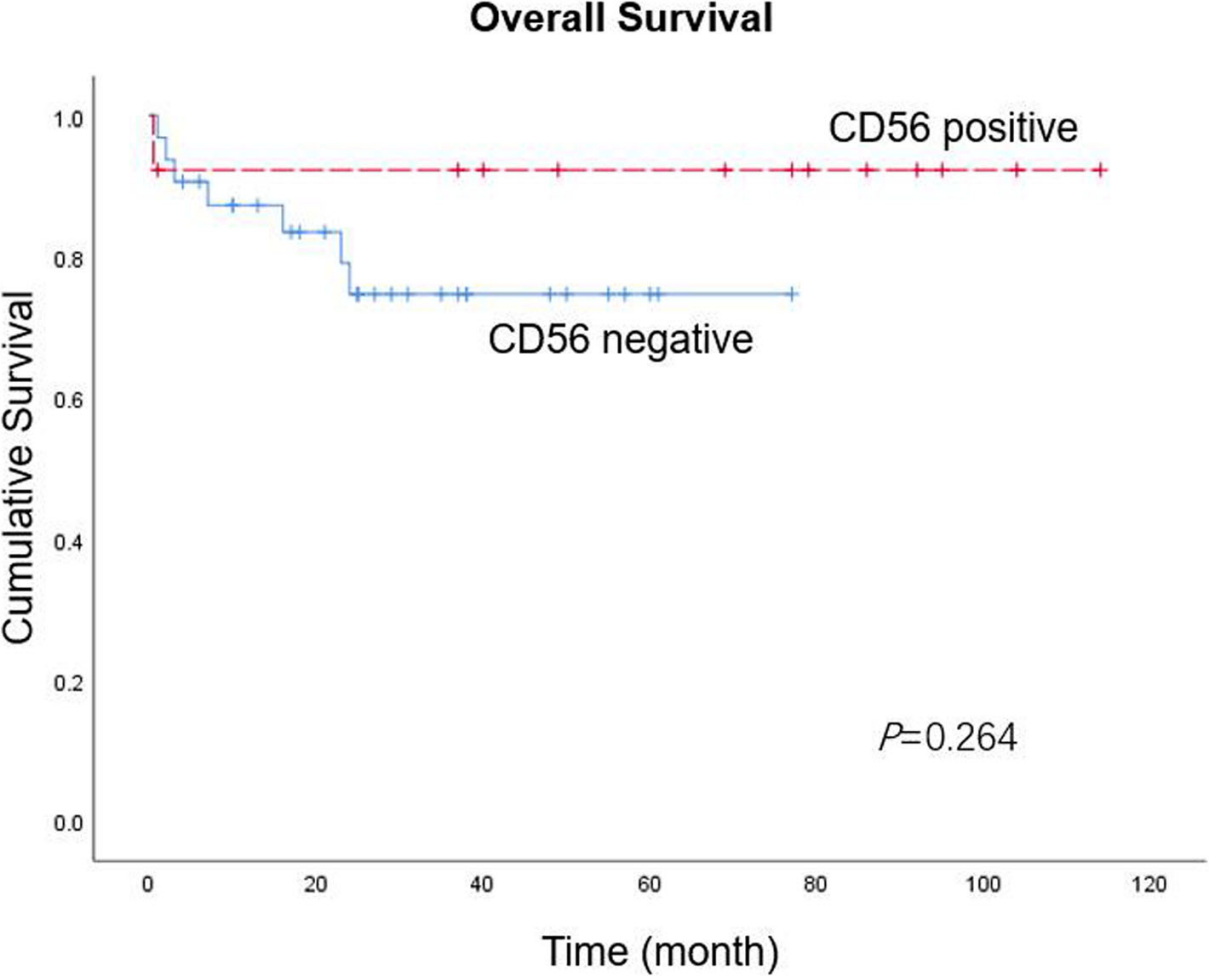
Kaplan-Meier survival curve of patients with ALK+ ALCLs. The 3-year OS rate in patients with uniform CD56 expression was 92.0%, compared with 73.0% in those with CD56 negative expression. However, there was no significant difference in OS between the two groups

The treatment and prognosis of all cases are shown in Table 1. All of the CD56+ ALK+ sALCL patients received systemic chemotherapy, except one patient with unavailable treatment information. The CHOP regimen (cyclophosphamide, doxorubicin, vincristine and prednisone) was the most frequently employed, and four patients received subsequent radiotherapy. The follow-up information was available for 12 patients, with a median follow-up time of 77 months (range, 0.5–114 months). The 3-year OS rate was 92.0%, which was better than that of CD56- ALK+ counterparts (73.0%). Although the Kaplan-Meier curve looked promising, there appeared to be no significant difference in OS between CD56+ and CD56- ALK+ ALCLs (*p* = 0.264) (Fig. 3).

## Discussion

ALCL with diffuse CD56 expression has rarely been documented. In a study of 140 sALCLs, CD56 expression was found in 17.9% of cases [5]. In another series, 15% of 75 pediatric ALCLs expressed CD56, including both systemic and primary cutaneous cases [12]. The incidence in our study, either sALCLs or pcALCLs, was much lower than that reported previously, which might be partially due to the different criteria for CD56 positivity. The frequency of CD56 expression in ALCLs might be underestimated given that CD56 was not always evaluated in each case of ALCL.

It has been stated that CD56+ hematologic malignancies have a remarkable predilection for extranodal involvement [7]. Consistently, our results demonstrated an extranodal predominance of CD56+ ALCLs. Notably, the upper aerodigestive tract was not infrequently involved by CD56+ ALCL in our study (*n* = 3), making the diagnosis confusing and challenging, given that this region was more commonly affected by ENKTL, with CD56 expression as its most consistent feature. Interestingly, uniform CD56 expression was frequent in our cases with breast ALCL, despite a very small case number. ALCL involving the breast is exceedingly rare, and CD56+ cases have never been documented [13]. It is of interest to encourage further investigation into the possible location predilection of CD56+ ALCL.

The description of the histological features of CD56+ ALCL was limited. d’Amore et al. indicated that CD56+ was more frequently seen in cases composed of small- or medium-sized tumor cells than in classic ALCLs [12]. Somewhat consistently, 2 of our cases were of small cell variant. In addition, Boudova et al. described a primary cutaneous CD56+ ALCL of the histiocyte and neutrophil-rich subtype, which displayed a dissolute growth pattern of neoplastic cells marked by an inflammatory background [10]. These findings extended the histological spectrum of CD56+ ALCL.

One noteworthy finding in the current study was the diffuse CD56 expression in a few ALCL cases. Nevertheless, the value of CD56 expression in the prediction and prognosis of ALCL appears to be poorly illustrated and complicated. CD56 expression in hematologic malignancies frequently predicts a poor outcome, despite aggressive treatment [7]. However, its prognostic significance in ALCL remains contradictory. Suzuki et al. demonstrated that CD56 expression in ALCL was associated with a poor prognosis, while their study had an ill-defined mixture of adult and pediatric, ALK-positive and ALK-negative cases, as Dunphy et al. concluded [5, 7]. In contrast, aberrant CD56 expression might not affect the clinical behavior and outcome of pcALCLs according to several sporadic case reports, which behaved largely in an indolent way similar to other pcALCLs and much better than other CD56+ lymphomas involving the skin [7, 10, 14–17]. Unfortunately, although the Kaplan-Meier curve in the current study looked promising, there appeared to be no significant difference in OS between CD56+ and CD56- ALK+ sALCLs, which might be due to the small case series of CD56+ cases. Therefore, the prognostic relevance of CD56 in ALCLs deserves to be further investigated with a much larger series.

Owing to the similarities in morphology and/or immunophenotype, CD56+ ALCL should be distinguished from other types of lymphomas, among which the most confusing is ENKTL with CD56 staining. There are great overlaps in the morphology, immunophenotype and even molecular manifestations between the two entities, resulting in a potential diagnostic dilemma. In detail, ENKTLs mainly composed of pleomorphic large cells with diffuse CD30 expression bear a striking resemblance to ALCL; conversely, ALCLs with intensive CD56 staining might also be misdiagnosed as ENTKL, especially those involving the nasal cavity and the surrounding areas with negative ALK staining or without ALK investigation. EBV infection and ALK status appear to be the most helpful distinguishing feature, although occasional ALCL cases were also reported to harbor EBV mRNA expression [18–20]. Distinguishing ENKTL and ALCL coexpressing CD56 and CD30 is not of simple academic interest, but has great clinical relevance owing to the different therapeutic approaches and prognoses of these two diseases. Thus, increasing the awareness and being familiar with this group of ALCL with unusual phenotype is very important. CD56+ ALCL might also mimic other tumors morphologically, including plasma cell neoplasms and malignant melanoma. Combined applications of careful histological examination, immunohistochemical staining and molecular detection are of great value to facilitate differential diagnosis.

## Conclusions

In conclusion, uniform CD56 expression is an unusual condition in ALCL and predominantly occurs in ALK+ ALCLs at an early stage with a predilection of extranodal site involvement. Increased awareness of the potential occurrence and a better understanding of CD56 expression in ALCL is essential for avoiding both diagnostic and therapeutic pitfalls. Further studies are encouraged to fully elucidate the significance of CD56 expression in ALCL, including its value in prediction and prognosis.

## Data Availability

The data are available upon request from the corresponding author: leexiaoqiu@hotmail.com.

## Abbreviations

ALCL: Anaplastic large cell lymphoma
EBV: Epstein-Barr virus
TCR: T-cell receptor
WHO: World Health Organization
ENKTL: Extranodal NK/T-cell lymphoma, nasal type
EBER: EBV-encoded small nuclear early region
PCR: Polymerase chain reaction; OS: overall survival
